# Bovine serum albumin-protected copper nanoclusters as a label-free biosensor for the discrimination of bacterial strains

**DOI:** 10.1038/s41598-025-25219-6

**Published:** 2025-11-18

**Authors:** Saad Megahed, Noha El Salakawy, Wael Mamdouh

**Affiliations:** 1 Department of Chemistry, School of Sciences and Engineering, The American University in Cairo (AUC), New Cairo, 11835 Egypt; 2 Physics Department, Faculty of Science, Al-Azhar University, Cairo, 11884 Egypt

**Keywords:** Copper nanoclusters, Selective label-free biosensor, Peroxidase-like activity, Bacterial detection, Microbiology, Chemistry, Nanoscience and technology

## Abstract

The development of biosensors for infectious diseases is crucial, especially in the post pandemic era. Point-of-care (POC) devices are highly needed in developing countries to provide quick and cost-effective results. Nanoparticles with enzymatic-like activity, also known as nanozymes, show great promise for diagnostic applications and have recently gained significant attention. In this study, bovine serum albumin (BSA)-protected copper nanoclusters (Cu NCs) with enzymatic-like characteristics were created and analysed for their intrinsic enzymatic-like activity, physicochemical properties, and colloidal stability. The Cu NCs were synthesized via the use of BSA as a capping and reducing agent to ensure their biocompatibility and colloidal stability. Various techniques, such as UV‒Vis spectroscopy, photoluminescence (PL), dynamic light scattering (DLS), elemental analysis, and transmission electron microscopy (TEM), have been used to characterize the prepared Cu NCs. The Cu NCs demonstrated oxidase- and peroxidase-like activity by catalyzing the oxidation of o-phenylenediamine (OPD) into 2,3-diaminophenazine (DAP), the oxOPD product. Showing higher oxidase-like activity by 1.6x compared to the peroxidase-like activity at the same substrate concentration. Their catalytic properties were utilized to detect two common bacteria, gram-negative *Escherichia coli* (*E. coli*) and gram-positive *Staphylococcus aureus* (*S. aureus*), demonstrating a differential label-free detection of different strains at concentrations higher than 10^5^ CFU mL^− 1^. This study introduces a promising and affordable application for a label-free biosensor used in detecting infectious diseases.

**Supplementary Information:**

The online version contains supplementary material available at 10.1038/s41598-025-25219-6.

## Introduction

In recent years, the prompt and fast development of materials science has led to the design and introduction of several types of nanoscale materials with unique anti-bacterial^1^, anti-cancer^2^, optical^3^, catalytic^4,5^, and enzyme-like^4–6^ characteristics, resulting in practical applications of these nanostructures in different areas, for instance, sensing and detection^7^, photocatalysis^8^, chemical catalysis^9^, nanodrug development^10^, water treatment^11^, and point of care detection^12^ Metal nanoclusters have recently attracted considerable interest due to their distinct properties and wide-ranging applications. Unlike individual atoms, molecules, or bulk materials, nanoclusters exhibit unique characteristics, making them highly promising for use in biosensing, bioimaging, artificial enzyme design, and optoelectronic devices^3,4,13–17^.

Metallic nanoclusters with a size regime between nanoparticles and molecules start to lose their plasmonic bands while offering unique optical properties on the basis of their size and composition. Owing to their small size, the electronic transition between their energy levels leads to the fluorescence property of the nanoclusters. For many years, the optical properties of nanoclusters have been the main focus of their applications; however, little attention has been given to their catalytic capability. Among these nanoclusters, extensive research has focused on gold and silver nanoclusters because of their known plasmonic properties and large-scale applications in different fields^1,18–21^.

Compared with those of gold and silver nanoclusters, the structure and properties of copper nanoclusters (Cu NCs) are less described in the literature. Notably, Cu NCs have been synthesized via different methods and approaches, including DNA, RNA, polymers, proteins, and thiols, which are used to control their nucleation and growth^22–25^. The optical properties - the fluorescence emission - of synthesized Cu NCs can be tuned primarily by adjusting the protective ligand and controlling their size. For instance, Li et al. (2021) demonstrated that varying the reaction conditions while employing the same stabilizer-chicken egg white-yielded Cu NCs emitting in different colors, such as green, yellow, orange, and red-emitting Cu NCs^26–29^.

Nanozymes have recently attracted considerable interest in research because of their potential as alternative materials to natural enzymes, which present multiple challenges, from their sensitivity to their stability in different environments^30–35^. Nanozymes have been introduced in the first report of Gao et al. in 2007; they exhibit peroxidase-like (POD-like) activity and are thought to be biologically and chemically inert. Since then, several reports have been published on the use of different classes of nanomaterials, including noble metals, metal oxides, and polymeric nanomaterials^4,36–42^. Certain NZs exhibit multifunctional oxidoreductase-like activities, including catalase (CAT), oxidase (OX), glucose oxidase (GOD), superoxide dismutase (SOD), glutathione peroxidase (GPx), and peroxidase (POD) behaviors, among others^6^. Their catalytic effects are categorized as either antioxidants (scavenging reactive oxygen species, ROS) or pro-oxidants (generating highly reactive intermediates, HRI), depending on their interaction with substrates and redox potential. The pro-oxidative POD-like activity of NZs produces potent oxygen radicals, such as hydroxyl radicals, which can degrade organic molecules, including dyes and proteins^5,43^. This property has been leveraged for environmental applications, such as pollutant breakdown, and biomedical uses, such as targeted protein oxidation and chemodynamic therapy (CDT).

Copper has been reported as a potential nanozyme in different reports related to glucose detection, protein detection, pH sensing, bioimaging, and infection detection^17,34,44–49^. Cu is an abundant, inexpensive, and highly reactive metal compared with other noble metals. Despite the difficulty in controlling the size and reactivity of Cu NCs, recent reports have successfully illustrated the synthesis of Cu NCs with good stability. Zhao et al. used transferrin as a template and reducing agent to study the overexpression of its receptor in cancer cells^45^. In addition, Miao et al. (2018) introduced BSA-Cu NCs as colorimetric and fluorescent probes to detect bilirubin^50^. Moreover, Han et al. 2024 utilized the POD activity of Cu NCs for glucose detection in addition to other applications in sensing, diagnostics, and bioimaging probes^51–55^.

Different approaches have been introduced to synthesize more stable Cu NCs via the use of small ligands and biomolecules. Notably, controlling the ligand, pH, temperature, and reaction conditions helps in tuning the final product of the Cu NCs in terms of size and optical properties. The advantage of using biomolecules as capping and reducing agents in the process of Cu NC preparation is that they retain the stability and biocompatibility of the Cu NCs. The fluorescence property of the Cu NCs, along with their biocompatibility, gives them a particular advantage in the bioimaging field. Moreover, the catalytic and enzyme-like activity of the Cu NCs should also receive more attention and be combined with their optical properties to provide multiplex NCs for biomedical applications.

In the present work, we successfully synthesized protein-protected Cu NCs that were stable in different environments. The BSA-Cu NCs showed enzyme-like activity, such as oxidase-like and peroxidase-like activity, with different kinetics. The BSA-Cu NCs emitted green fluorescence at 460 nm and were highly biocompatible with gram-positive and gram-negative bacterial strains. In addition to their catalytic activity, Cu NCs represent a colorimetric way to distinguish different bacterial strains on the basis of their ability to oxidize the OPD substrate.

## Results and discussion

### Physicochemical characterization

The synthesis of the BSA-protected Cu NCs was carried out according to a previously published protocol with slight modifications (further details are described in the SI). At neutral pH, the carboxylate groups of BSA formed a complex with Cu^2+^, forming a viscous fluid. The pH increased when NaOH was added to the mixture to further increase the solubility to obtain a clear purple solution. Mild reducing agents reduce the Cu^2+^ ions into metallic copper; as BSA is a weak reducing agent, H_2_O_2_ has been added to further facilitate the reduction of the Cu^2+^. The color of the solution turned brown, indicating the formation of the Cu NCs (Figure S1)^56,57^.

The thiols in BSA are highly essential for their interaction with Cu^2+^ to form Cu–S bonds, resulting in a protective layer of BSA surrounding the surface of the Cu NCs. This helps protect the NCs from oxidation and keeps them stable^57^, as will be shown in the next section.

**Fig. 1:**
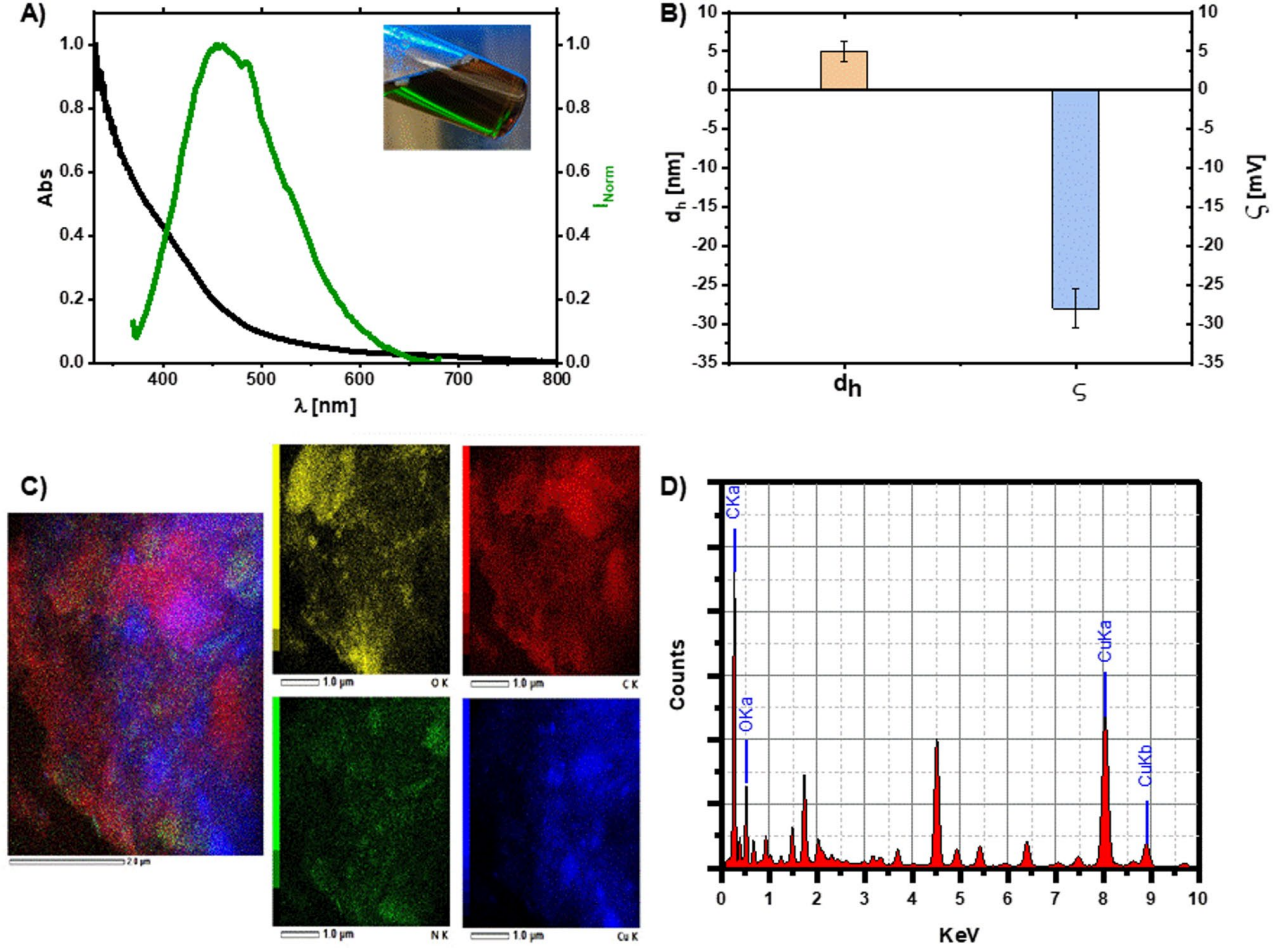
Characterization of the prepared Cu NCs. (**A**) UV‒Vis absorption (black) and photoluminescence (PL) spectra (green), inset = Cu NCs exposed to UV light showing the green fluorescence of the Cu NCs, (**B**) hydrodynamic diameter d_h_ and ζ-potential, (**C**) EDS‒TEM mapping of the NCs showing the distributions of different elements, such as O (yellow), C (red), N (green), and Cu (blue), and (**D**) elemental analysis of the NCs.

The optical properties of the as-prepared Cu NCs were studied via UV‒Vis spectroscopy and a PL spectrophotometer (Fig. 1A). The absorbance spectrum of the Cu NCs revealed a smooth exponential increase in the absorbance at higher energies, below 400 nm, which is characteristic of cluster formation^56^. Comparing the Cu NCs synthesized in the presence of H_2_O_2_ with those synthesized without H_2_O_2_ facilitates the formation of Cu NCs. As we can see from their absorption spectrum when H_2_O_2_ was used, the absorption peak of any residual from the Cu precursors was absent compared with that of the sample prepared without H_2_O_2,_ Figure S2. This agrees with a previous study reported by Chan Wang et al., where they used BSA as a protecting agent to form Cu NCs, and the XPS analysis of the prepared BSA-Cu NCs revealed the oxidation state of Cu (0)^58^.

The optical properties at such small sizes were affected mainly by quantum confinement; thus, according to the core size of the Cu NCs, they were expected to have distinct optical properties. The PL spectrum of the Cu NCs reached a maximum at 460 nm with intense green fluorescence, as shown in the inset image in Fig. 1A. This makes the prepared clusters good candidates for biological imaging because their surface is protected with BSA, which is a highly biocompatible surface ligand.

As the emission properties of the Cu NCs were size-dependent, they were also affected by the concentration of the ligands. Reza and co-workers reported that higher concentrations result in larger and weakly emissive NCs, whereas at moderate concentrations, brighter clusters were formed^56^. The smaller the clusters were, the higher the energy of the emission. Therefore, in the present green-emissive Cu NCs, we expect a smaller size of the NCs. This has been proven by measuring their hydrodynamic size via DLS, as shown in Fig. 1B. The size distribution of the Cu NCs was 5 ± 1.3 nm, on the basis of the number distribution, Figure S4 and observing their core size via TEM was quite difficult, as the clusters tended to agglomerate when they were dried on the grid. The surface charge of the NCs, as determined by measuring their zeta potential ζ, was – 28 ± 2.5 mV. This indicated good colloidal stability of the prepared Cu NCs, assuming that the stability was governed by both electrostatic repulsion, which was achieved via measurement of their surface charge, and steric hindrance, which was assisted by the presence of a thick layer of the BSA protein surrounding the Cu NCs.

Energy dispersive spectroscopy (EDS) of the NCs confirmed the presence of the elements corresponding to the Cu NCs and the BSA protein, as shown in Fig. 1C and D. In addition, the graphs in Fig. 1C show their homogenous distribution throughout the sample. The content of BSA was almost 4-fold greater than the Cu content, as obtained from the EDS spectral analyses in Figure S5 and Table S1. This agrees with the initial concentration ratio used to prepare the Cu NCs and to obtain more stable NCs by having more content from the BSA layer on the Cu NCs. The capping of BSA to the Cu NCs has been further supported by the FT-IR spectra of the BSA and BSA-Cu NCs, Figure S3.

### Stability of the nanozyme

The colloidal stability of the Cu NCs was tested against different media, including Milli Q water, pH 4, 7.4, and 11. The NCs were quite stable against different media (Figures S6 to S10). The lack of significant changes in their optical properties indicated that no further changes in the structure or size of the NCs occurred upon exposure to different media. Notably, the NCs showed solution turbidity upon exposure to a highly acidic medium, which was expected, as the working pH was below the isoelectric point of BSA, as shown in Figure S6:S8. However, such behavior was reversible upon changing the pH of the solvent, where the NCs recovered their properties. The pH-responsive behavior of the BSA-protected Cu NCs agreed with that of different Cu NCs prepared with proteins as capping agents, such as Cu NCs prepared from chicken egg white CEW, which showed pH-responsive properties, most likely due to the conformational changes in the ovalbumin protein in CEW with pH variation. These Cu NCs showed intense green emissions, similar to the Cu NCs presented in the current study, under strongly basic conditions, whereas under lower or acidic conditions, the PL was quenched as a result of NC precipitation^56,59^.

Moreover, we examined the colloidal stability of the Cu NCs in LB medium. As shown in Figures S9 and S10, we also observed good stability with no significant change in their optical properties, as supported by their UV‒Vis spectra or their hydrodynamic sizes. These results confirmed the good stability of the Cu NCs upon incubation or exposure to different media.

### Oxidase and peroxidase-like activity

**Fig. 2:**
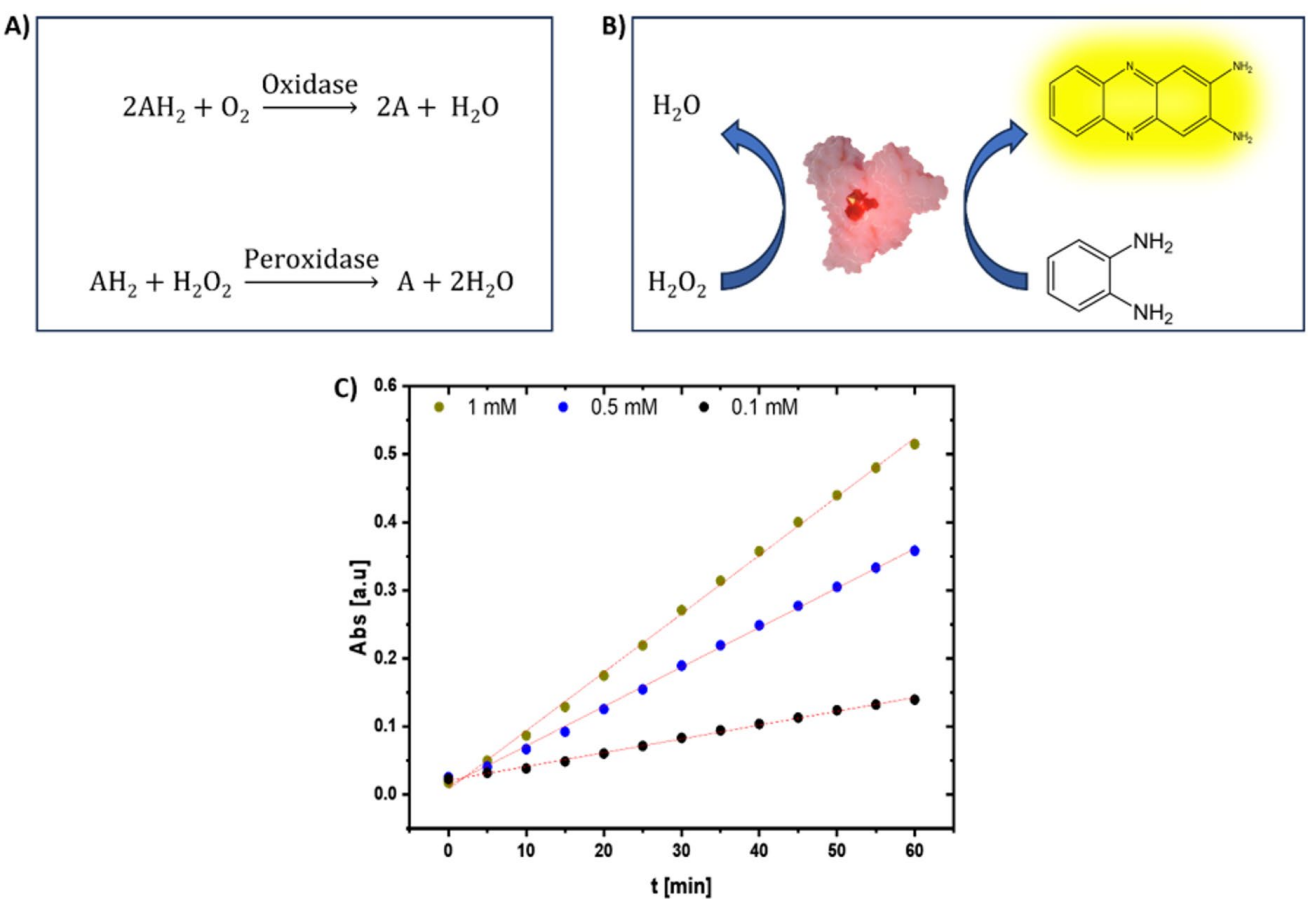
Oxidase-like activity of the Cu NCs. (**A**) Scheme illustrating the reactions of OX and the POD natural enzyme. (**B**) Schematic illustration of the oxidase-like activity of the Cu NCs in the oxidation of OPD (no color) into DAP (yellow color) and their POD-like activity. (**C**) Evolution of oxOPD (DAP) absorption at λ = 425 nm for 1 h. at different concentrations of 1 mM (green), 0.5 mM (blue), and 0.1 mM (black) in the presence of Cu NCs.

The artificial oxidase (OX) and peroxidase (POD)-like activities of the NCs were evaluated against the ortho-phenylenediamine (OPD) substrate. The catalytic activity of the NCs was traced through their ability to oxidize OPD (colorless) into 2,3-diaminophenazine (DAP) (yellow). The oxidation process was traced via a UV‒Vis spectrophotometer by recording the evolution of the absorption spectra at λ = 425 nm, the characteristic band of DAP (ε = 16 300 M^− 1^ cm^− 1^). Figure 2A, and 2B.

The oxidase- and peroxidase-like activities of the Cu NCs were investigated against OPD as a substrate without and with H_2_O_2_, respectively. The enzymatic activity was recorded for one hour by monitoring the evolution of the DAP peak at 425 nm every 5 min (Figure S11 and S12). By recording the evolution of the DAP peak at different concentrations of OPD, we can compare the catalytic dynamics of the Cu NCs at different concentrations. Figure 2C shows the growth of the absorption peak of DAP at λ = 425 nm at different concentrations over the recorded period. By increasing the concentration of OPD, a faster oxidation rate by the Cu NCs was observed.

Moreover, we investigated the peroxidase-like activity of the Cu NCs against OPD in the presence of H_2_O_2_. The obtained results showed a similar trend to that of the oxidation test of OPD, whereby increasing the concentration of OPD, we observed a faster rate of product formation. The adsorption of H_2_O_2_ onto the surface of the Cu NCs led to the breaking of the $\:\text{O}-\text{O}$ bond of H_2_O_2_ into two OH^**·**^ radicals, as reported elsewhere, which caused the catalytic activity of the Cu NCs, namely, their POD-like activity^36,60,61^. Interestingly, the Cu NCs showed efficient oxidase-like activity compared with their peroxidase-like activity. In the concentration range tested, the rate of oxidation of the substrate by the Cu NCs exhibited a linear stepwise increase compared with its peroxidase-like activity. In the latter case, the oxidation rate was increased to 0.5 mM, resulting in a plateau-like behavior. Therefore, at higher concentrations of OPD, there was no further increase in the oxidation rate in the presence of H_2_O_2_. For example, at 1 mM OPD, the oxidation rate of the substrate based on the OX activity of the Cu NCs was higher than the peroxidase-like activity by 1.6-fold. This difference in the catalytic activity in the presence of H_2_O_2_ could be due to the simultaneous catalase and peroxidase-like activities of the Cu NCs. The competitive effect of both enzymatic-like activities on the surface of the Cu NCs leads to different kinetics. It seems the NCs prefer to consume the H_2_O_2_ in the CAT-like pathway rather than in the POD-like pathway. Therefore, we have observed higher oxidative activity in the absence of H_2_O_2_. This was observed by the formation of bubbles inside the solution while H_2_O_2_ was present. Notably, at concentrations higher than 1 mM, the oxidation rate started following a linear behavior within the first 20 min of the reaction, which was similar to that at 1 mM, and then the rate decreased. This observation indicated that the substrate was almost consumed in the first 20 min of the reaction at higher concentrations.

### Cu NCs and H_2_O_2_ biocompatibility

**Fig. 3:**
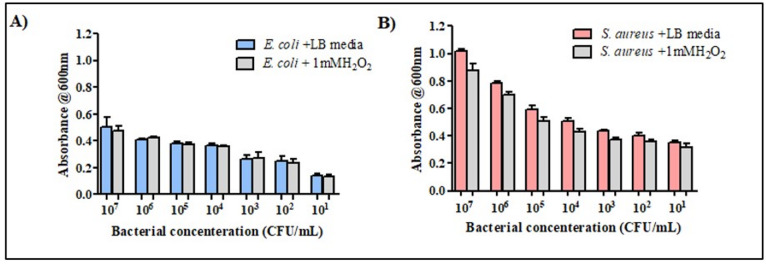
The effect of 1 mM hydrogen peroxide on the growth of; (**A**) *E. coli* and (**B**) *S. aureus* after 24 h, respectively.

**Fig. 4:**
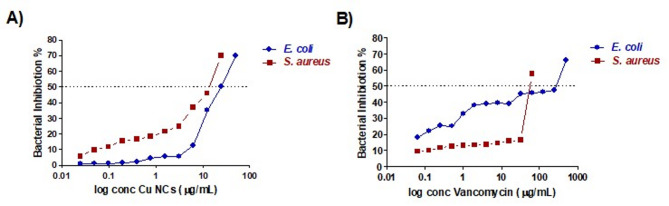
Minimum inhibitory concentration of (**A**) Cu NCs (µg/mL) and (**B**) Vancomycin (µg/mL) against *E. coli* (blue) and *S. aureus* (red).

The biocompatibility of the Cu NCs and the bactericidal effects of the working concentrations of H_2_O_2_ were tested before the biosensing experiment against two different bacterial strains, *E. coli* and *S. aureus*. The effects of H_2_O_2_ have been tested against various concentrations of bacterial strains ranging from 10 to 10^7^ CFU/mL. The inhibitory effect of H_2_O_2_ on the growth of the bacterial strains was negligible, and there was no bactericidal effect on either strain, as shown in Fig. 3. Moreover, the 50% bacterial inhibition of the Cu NCs was evaluated against both strains (results shown in Fig. 4), and the IC_50_ values were determined (Table S2).

### Selective colorimetric biosensing

The biocompatibility and catalytic properties of the prepared Cu NCs offer many benefits and applications in terms of bioimaging, diagnostic, and biosensing. In the present study, we investigated the differential biosensing applications of Cu NCs, where we tested their catalytic activity, converting OPD into oxOPD, in the presence of two different bacterial strains. There are different approaches for optical detection, such as fluorescence or absorbance, and in the present study, we traced the change in the absorbance of the oxidized product of OPD.

**Fig. 5:**
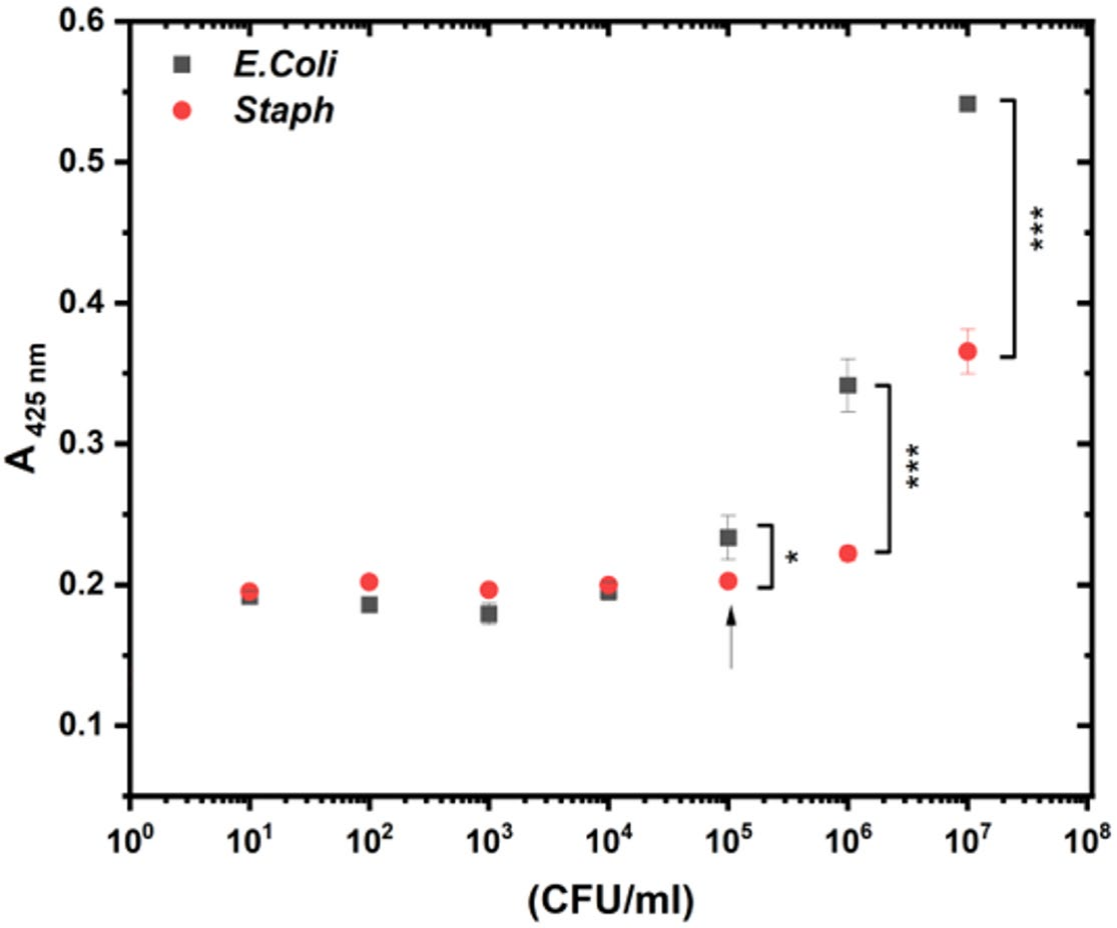
Bacterial-selective biosensing of Cu NCs against two different bacterial strains (*E. coli* and *S. aureus*). The absorption of the oxOPD at $\:\lambda\:=425\:nm$ using OPD and H_2_O_2_ (1 mM) with 12 µg/mL Cu NCs.

The absorbance of oxOPD at λ = 425 nm for both strains presented the same baseline at lower concentrations of bacteria up to 10^5^ CFU/mL, where from this point, a significant response of the gram-negative bacteria compared with the gram-positive strain was observed. Beyond this threshold, at 10^5^ CFU/mL, the response of *E. coli* was 1.5-fold greater than that of *S. aureus*, as shown in Fig. 5. The oxidation of OPD was significantly enhanced in the presence of *E. coli*, which was easily detected by tracing the absorbance of the sample. Nevertheless, a detectable absorbance percentage was observed at a low bacterial density (10^1^ CFU/mL), with the absorbance increasing as the bacterial density increased. A significant difference in absorbance was observed at a bacterial density of 10^5^ CFU/mL (P value < 0.05), reaching its maximum at 10^7^ (P value < 0.001). No noticeable difference was observed upon doubling the concentration of Cu NCs, highlighting the ability of low concentrations of Cu NCs to detect bacteria at very low densities. Furthermore, the selectivity of the Cu NCs in differentiating between *E. coli* and *S. aureus* was noted, as shown in Figure S13; A and B.

Additionally, Linear response was detected as absorbance increased with bacterial concentration. The linear range for *E. coli* was identified between 10^3^ and 10^8^ CFU/mL, while for *S. aureus* it ranged from 10^5^ to 10^8^ CFU/mL. (Figure S13 and S14)

The limits of detection (LOD) were measured as 10^2^ CFU/mL for *E. coli* and 10^3^ CFU/mL for *S. aureus* after 30 min. The limit of quantification (LOQ) was also measured as 10^3^ CFU/mL for *E. coli* and 10^4^ CFU/mL for *S. aureus* after the same duration.

In a previous study by Deng et al., CuO nanoparticles (NPs) were used to distinguish between gram-positive and gram-negative bacteria by tracing the fluorescence of the oxOPD. With different limits of detection LOD of several Gram-negative bacteria strains as follows: 2 CFU mL^− 1^ for *E. coli*, 4.1 × 10^3^ CFU mL^− 1^ for *S. typhimurium*, and 1.6 × 10^4^ CFU mL^− 1^ for *P. aeruginosa*. The authors reported a different phenomenon than what we observed in the present study, where they reported that the presence of *E. coli* caused quenching of the fluorescence signal of the oxOPD. The authors speculated that the presence of bacteria inhibited the oxidase-like activity of the CuO NPs, which in turn led to a reduction in the fluorescence signal of oxOPD as a product of the oxidation effect of the CuO NPs^62^. In the present study, we observed a different scenario, where the oxidase-like activity of the Cu NCs did not change once they were incubated with different bacterial strains. This finding most likely indicates that the bacteria did not affect the oxidase-like activity of the Cu NCs, while they could influence their peroxidase-like activity differently by enhancing the formation of the free radicals, in which they oxidized OPD into its oxOPD. This increased degree of oxidation was significantly greater in gram-negative bacteria than in gram-positive bacteria. Thus, the oxidation of OPD in a linear response pattern provides a potential selective colorimetric detection probe for different bacterial strains. Notably, both *E. coli* and *S. aureus* are susceptible to Cu NCs; however, the extent and mechanism of damage may differ due to variations in their cell wall structures. *E. coli* may exhibit faster changes in membrane permeability, while the thicker peptidoglycan layer of *S. aureus* remains vulnerable to reactive oxygen species (ROS) and ion-mediated disruptions.

## Conclusion

Bovine serum albumin (BSA)-protected copper nanoclusters (Cu NCs) were successfully synthesized and characterized, demonstrating robust colloidal stability across varying media conditions. While these Cu NCs maintained stability under different pH levels, they exhibited reversible destabilization under acidic conditions. Dynamic light scattering (DLS) and photoluminescence (PL) analyses confirmed the retention of their optical properties and uniform size distribution. Given their green fluorescence emission, these Cu NCs hold potential as probes for cellular imaging applications.

Furthermore, the Cu NCs displayed enzyme-mimicking behavior, catalyzing the oxidation of OPD to its oxidized form, oxOPD, both in the presence and absence of hydrogen peroxide (H₂O₂), indicative of oxidase-like and peroxidase-like activities, respectively. Notably, their oxidase-like activity surpassed their peroxidase-like performance. Additionally, the biocompatibility of these Cu NCs, along with their label-free biosensing capability, enabled selective discrimination between *Escherichia coli* and *Staphylococcus aureus* by enhancing OPD substrate oxidation.

Despite these promising findings, certain limitations warrant further investigation. Future studies could expand the scope by evaluating the Cu NCs against a broader spectrum of bacterial strains and fungal species. Moreover, practical applications in food and water safety monitoring could be explored to assess real-world biosensing efficacy. Nevertheless, the current results underscore the potential of Cu NCs as cost-effective alternatives to conventional analytical instruments, particularly in biosensing and point-of-care (POC) diagnostic devices.

It is worth noting that several challenges may arise when testing the biosensor on real samples. One of these challenges is the presence of interfering substances, such as proteins and fats, which can lead to false signals and reduce the sensor’s effectiveness. Additionally, non-specific binding between different bacterial strains may occur. The accuracy of the biosensor can also be affected by changes in pH, salinity, or temperature of the tested samples. All of these factors should be taken into consideration when scaling up the biosensor for further investigation. Addressing these issues is essential for successfully implementing bacterial biosensors in real-world applications.

## Materials and methods

### Materials

Bovine serum albumin (BSA) (Fisher Scientific UK15561020), copper II sulfate pentahydrate (CuSO_4_.5H_2_O) purchased from Fisher Scientific UK(10627162), hydrogen peroxide (H_2_O_2_) (Sigma‒Aldrich Germany 822287), and sodium hydroxide (NaOH) were purchased from Sigma‒Aldrich Germany 221,465. Acetate buffer (pH 4) and phosphate-buffered saline (PBS, pH 7.4) were prepared in the laboratory, and ortho-phenylenediamine (OPD), Fisher Scientific 34,005, H_2_O_2_, and Milli-Q water were used throughout the experiments. The bacterial strains used were *Escherichia coli*, and *Staphylococcus aureus* cultured in Luria–Bertani (LB) broth media(Thermo Fisher Scientific J75852.30).

### Synthesis of Cu NCs

The BSA-protected Cu NCs were synthesized according to a previous protocol with slight modifications^57^. Briefly, 2 mL of CuSO_4_ (20 mM) was added to a BSA (15 mg/mL) aqueous solution at room temperature under vigorous stirring. The mixture was incubated for 5 min at RT, 0.15 mL of NaOH (1 M) was added to the mixture to adjust the pH of the mixture, and the color changed from turbid to clear violet. To facilitate the formation of the Cu NCs in a short time, 3 mL of H_2_O_2_ (0.1 M) was added, and the solution turned brown after the first mL was added (Figure S1). The mixture was left for 2 h at 55 °C to obtain the BSA-Cu NCs. The final product was washed with a dialysis bag and then kept at 4 °C for further experiments.

### Physicochemical characterization and stability of the Cu NCs

The optical properties of the Cu NCs were determined via a UV‒Vis spectrophotometer and a photoluminescence spectrophotometer (PL). The surface charge was measured via the ζ-potential, and the size distribution and elemental analysis were characterized via dynamic light scattering (DLS) and EDS-TEM mapping. The colloidal stability of the NCs was tested against different media and conditions via a UV‒vis spectrophotometer and DLS. The reader is referred to the supporting information file for further details.

### Bacterial sensing of Cu NCs using OPD as an indicator

The interaction between the Cu NCs and gram-negative and gram-positive bacteria (*Escherichia coli* (ATCC 25922) and *Staphylococcus aureus* (ATCC 25923), respectively) was tested with OPD as an indicator. H_2_O_2_ was added as an oxidizing agent to accelerate the interaction. In a 96-well microtiter plate, 100 µL of each bacterial strain at concentrations ranging from 10 to 10^7^ colony-forming units per milliliter (CFU/mL) was added. Then, 50 µL of Cu NCs at concentrations of 12 and 24 µg/mL, along with 20 µL of 1 mM H_2_O_2_, were added to the plate. The plates were then incubated for 30 min at 37 °C. After incubation, 50 µL of 10 mM OPD was added to the mixture, and the plates were incubated again for 30 min at 37 °C. The absorbance was measured at 425 nm via a Tecan Spectro plate reader (INFINITE M PLEX).

## Supplementary Information

Below is the link to the electronic supplementary material.

Supplementary Material 1

## Data Availability

The authors declare that the data supporting the findings of this study are available within the paper and its Supplementary Information files. Any raw data files are available from the corresponding author upon reasonable request.
