# Supplementary material for: Bovine serum albumin-protected copper nanoclusters as a label-free biosensor for the discrimination of bacterial strains
Source: Sci Rep. 2025 Nov 18;15:40374. doi: 10.1038/s41598-025-25219-6 (PMC12627415; doi:10.1038/s41598-025-25219-6)
Supplement: Supplementary file 1 — Supplementary Material 1 [file 41598_2025_25219_MOESM1_ESM.docx]

**Bovine Serum Albumin-Protected Copper Nanoclusters as a Label-Free Biosensor for the Discrimination of Bacterial Strains**

Contents

[S1. Synthesis of the copper NCs: 2](#_Toc209895222)

[S2. Characterization of the NCs: 2](#_Toc209895223)

[S2.1. Optical Characterization: 2](#_Toc209895224)

[S2.2. Hydrodynamic diameter and ζ-potential measurements: 4](#_Toc209895225)

[S2.3. The EDS Mapping of the Cu NCs: 5](#_Toc209895226)

[S3. Colloidal stability of the NCs: 6](#_Toc209895227)

[S3.1. Stability vs pH: 7](#_Toc209895228)

[S3.2. Stability vs LB medium: 9](#_Toc209895229)

[S4. Enzymatic-like activity of the NCs: 10](#_Toc209895230)

[S5. Bacterial sensing of Cu NCs 13](#_Toc209895231)

[S5.3. Quantitative antimicrobial activity of Cu NCs 13](#_Toc209895232)

[S5.4. Statistical Analysis 14](#_Toc209895233)

[S6. References 14](#_Toc209895234)

# S1. Synthesis of the copper NCs:

| 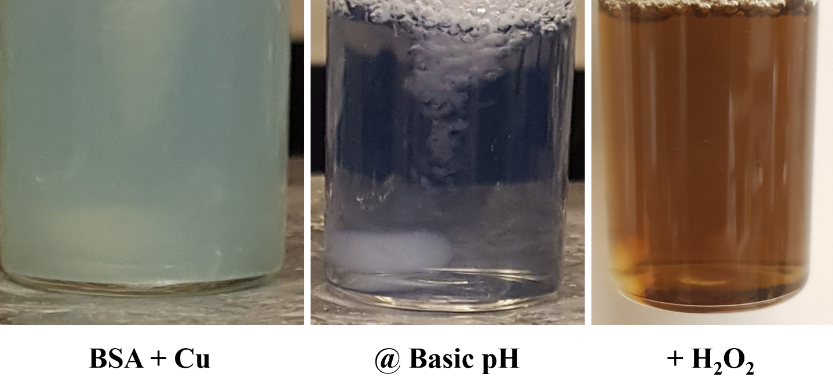  Figure S1 Synthesis process of the Cu NCs. |
| --- |

# S2. Characterization of the NCs:

The physico-chemical characterizations of the Cu NCs were carried out using different techniques such as UV-vis spectroscopy, photoluminescence spectrophotometer (PL), dynamic light scattering (DLS), ζ-potential, EDS-TEM mapping.

## S2.1. Optical Characterization:

The Cu NCs have been characterized using a UV-Vis spectrophotometer (UV Agilent Cary, UV-vis Compact mode) and a photoluminescence spectrophotometer PL. A suitable concentration of the NCs was loaded into a quartz cuvette (10 mm) and obtained spectrum as shown in the main text. In the case of measuring the PL of the NCs, the sample was excited at 350 nm, and the λ_max_ of the emission spectrum was at 460 nm. The Cu NCs prepared without adding the H_2_O_2_ show the existence of Cu precursors in their absorption peak, indicating not a full conversion or formation of Cu NCs. In addition, their PL spectrum is blue-shifted compared to the Cu NCs synthesized using H_2_O_2_.

Figure S2 UV-vis and PL spectra of the Cu NCs prepared w/o H_2_O_2_. The black arrow refers to the peak at 550 nm indicating the existence of CuSo_4_ residual and the inset shows the blue shift of the PL peak of the Cu NCs prepared w/o H_2_O_2_ (Blue) compared to that with H_2_O_2_ (Green).


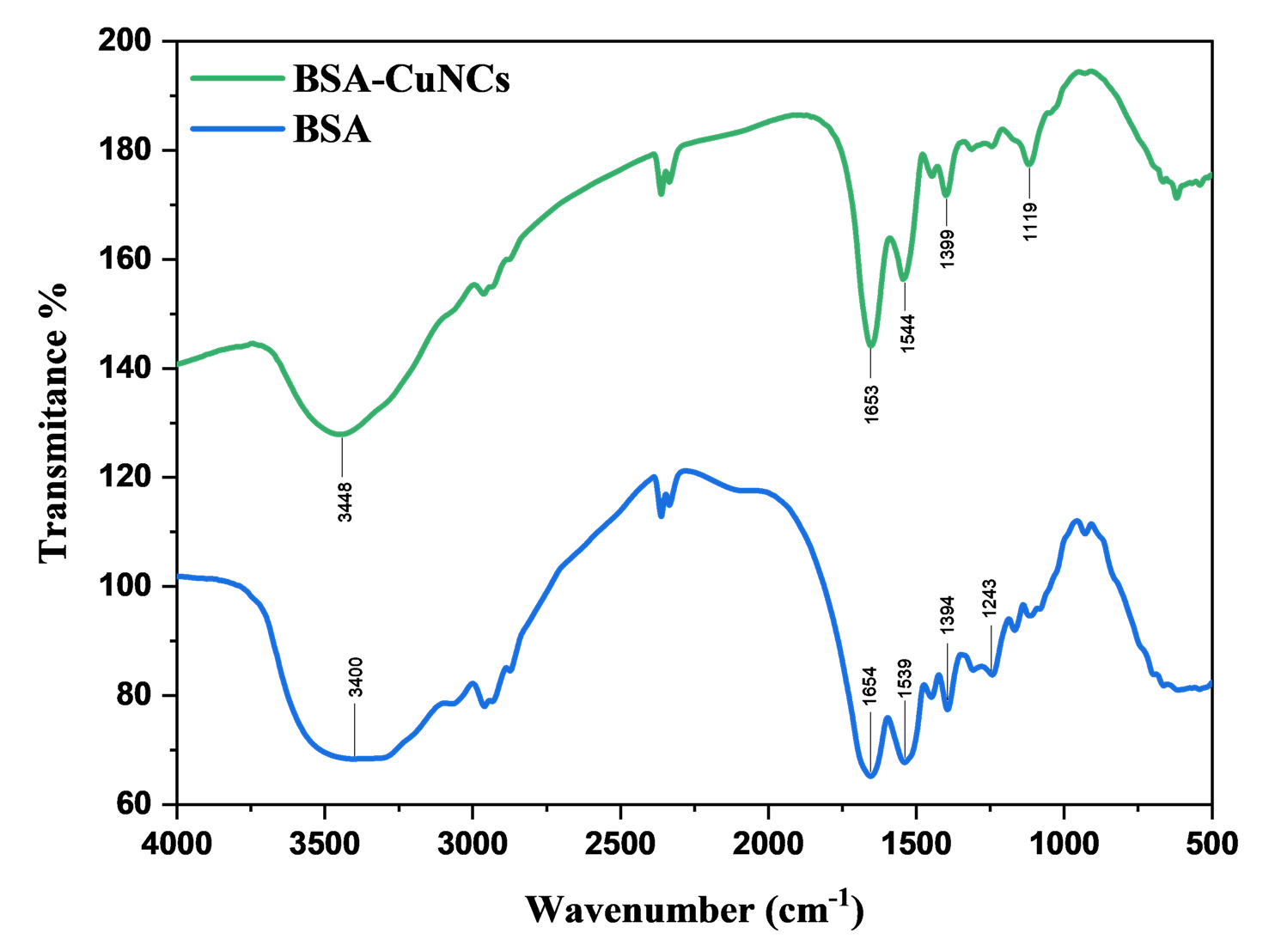


Figure S3 FT-IR spectra of BSA and BSA-Cu NCs. The IR peaks at 1653 cm^-1^ are assigned to amide I ($C=O$ stretching vibrations), 1539 cm^-1^ assigned to the amide II (coupling of bending vibrations of $N-H$ and stretching vibrations of $C-N$), and the peaks around 1394 cm^-1^ correspond to the carboxylate groups of the BSA.

## S2.2. Hydrodynamic diameter and ζ-potential measurements:

The hydrodynamic size and the zeta potential of the obtained NCs were evaluated using dynamics light scattering DLS (Malvern Nano-ZS90). Herein, we are showing the size distribution of the prepared Cu NCs based on number distribution, on the graph the mean value, and the standard deviation (out of three measurements). A histogram of the measurements for both the hydrodynamic size and the zeta potential is shown in the main text.


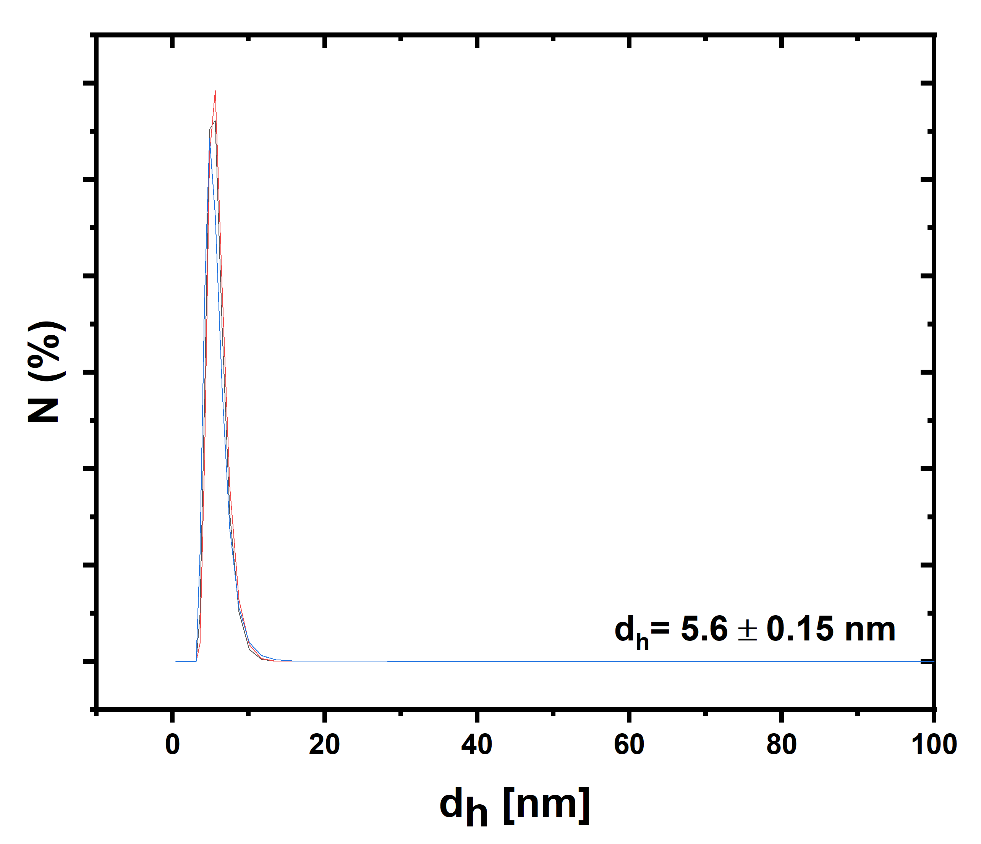


Figure S4 Hydrodynamic diameter distribution (based on number distribution) of the Cu NCs. Inset is the mean ± SD, of three measurements.

## S2.3. The EDS Mapping of the Cu NCs:

The distribution and elemental analysis of the NCs have been investigated using a transmission electron microscope (Jeol (JEM-2100 PLUS) at an accelerating voltage of 200kV). The energy dispersive X-ray spectroscopy (EDS) elemental mapping analysis together with scanning transmission electron microscopy (STEM) was performed on a JEM-2100 F (URP) instrument operating at 200 kV and equipped with a Dry SD30GV Detector.


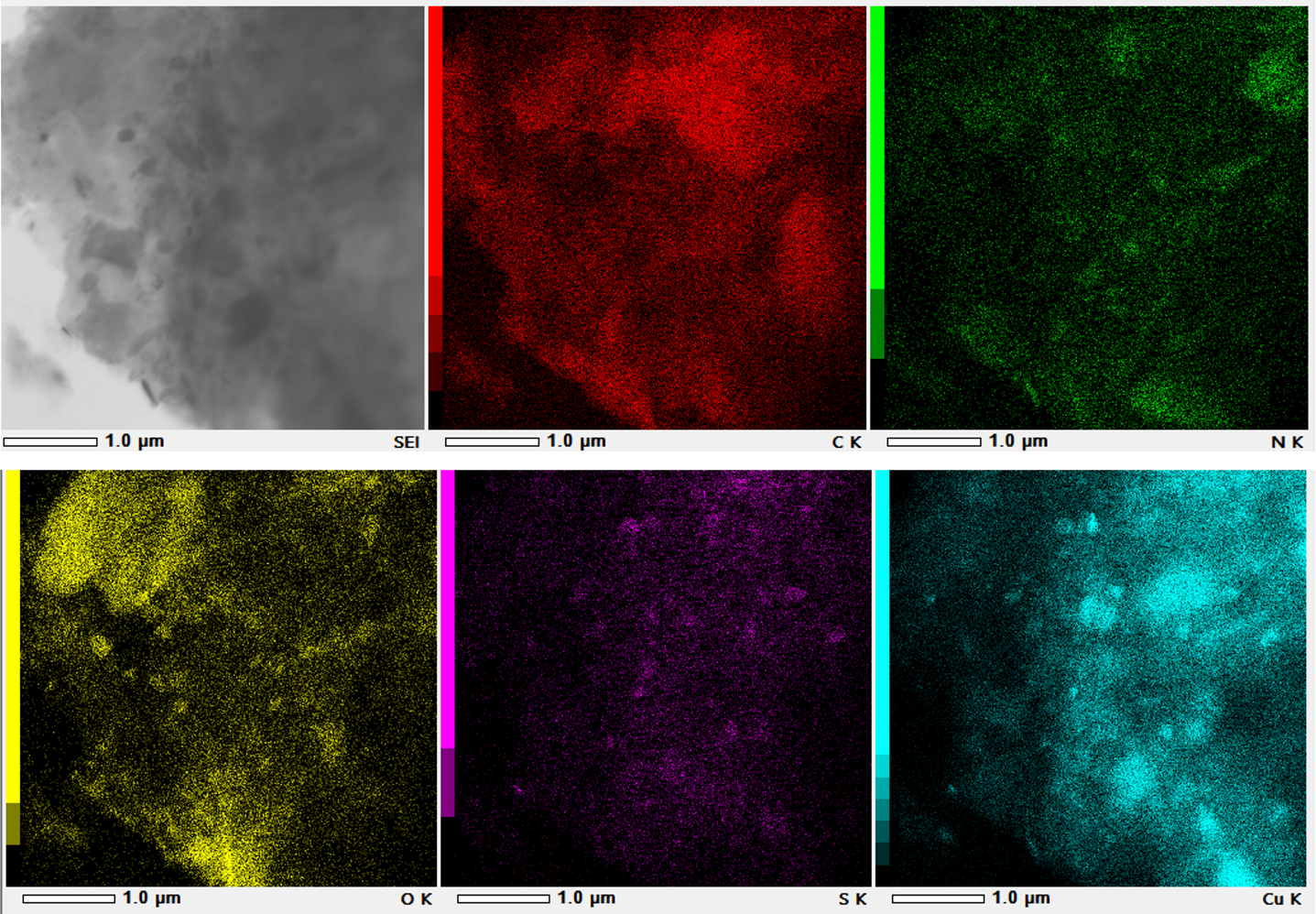


Figure S5 Elemental analysis and distribution of the Cu NCs showing the elements of C (red), N(green), O(yellow), S(purple), and Cu (gray).

Table S1 Elemental analysis of the Cu NCs as obtained using TEM-EDS mapping.

| Element | KeV | Mass% | Atom% |
| --- | --- | --- | --- |
| C | 0.277 | 56.69 | 71.38 |
| O | 0.525 | 24.7 | 23.35 |
| Cu | 8.04 | 17.28 | 4.11 |
| N | 0.392 | 0.87 | 0.94 |
| S | 2.307 | 0.46 | 0.22 |

# S3. Colloidal stability of the NCs:

The colloidal stability of the prepared Cu NCs has been tested versus different media. The colloidal stability was tested against different pH media and LB medium. The stability of the obtained NCs was evaluated based on their absorption and their DLS profile after incubation in different media for 0 and 24 h.

## S3.1. Stability vs pH:

The colloidal stability of the prepared Cu NCs has been tested against oxidation and different pH media. Both prepared samples have been tested, and both samples showed immediate instability against pH 4 as shown in the following figures. However, such instability was reversible by changing the pH of the medium the NCs dispersed again clearly. We have also measured their absorption spectra after incubation of 20 days which show quite stable against the different conditions.


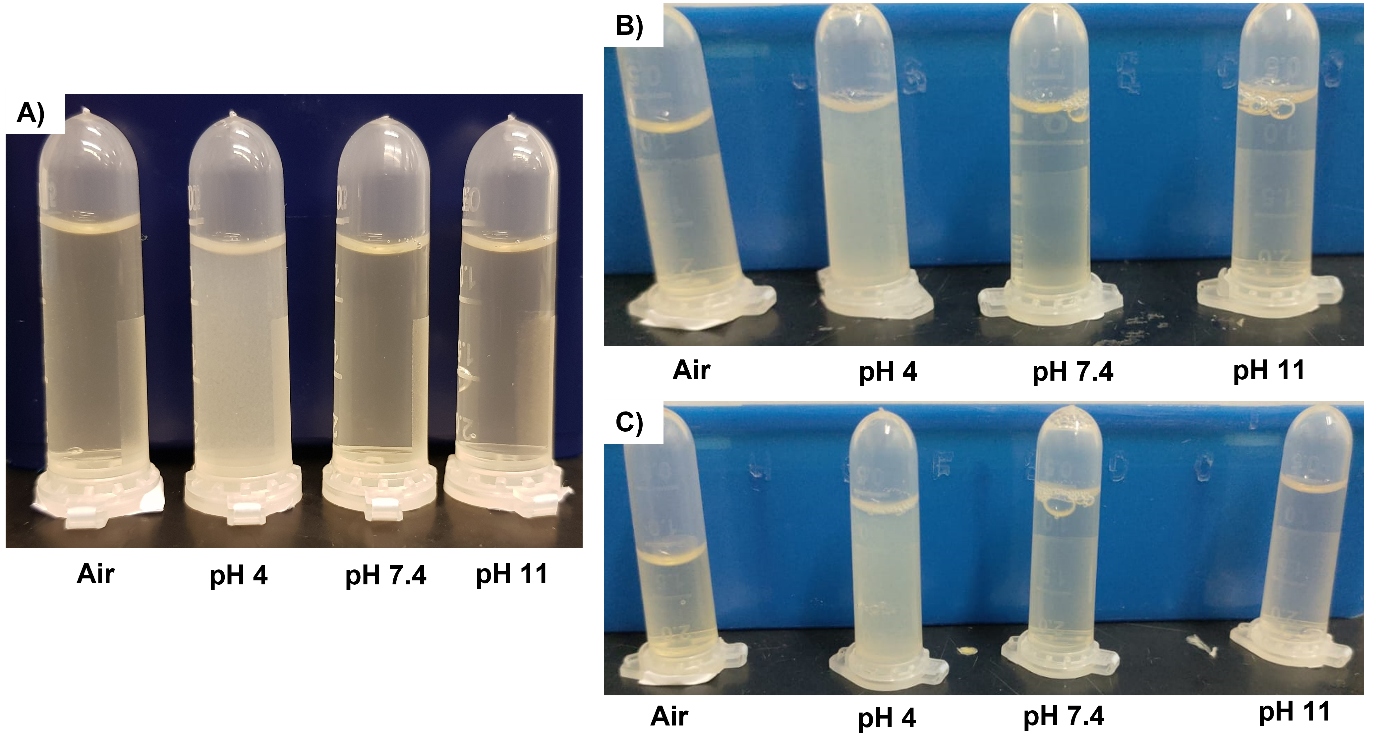


Figure S6 Colloidal stability of the Cu NCs vs oxidation and different pH media at different time points A) 0 h, B)24 h, and C)7 days.

Figure S7 UV-vis spectra of the Cu NCs against different pH media. The results have been taken after 20 days of incubation.

Figure S8 Hydrodynamic diameter (based on number distribution) of the Cu NCs vs pH at two different time points, 0 h and 24 h.

## S3.2. Stability vs LB medium:

In addition to evaluating the colloidal stability of the Cu NCs against different pH media, we have also examined their colloidal stability after incubation with LB broth medium at two different time points. The following graphs show the UV-vis spectra of the Cu NCs and their hydrodynamic diameter after incubation with LB medium at two different time points, 0 h, and 24 h. In brief, the absorption spectra showed no significant change upon the incubation with LB, so we did not observe any agglomeration or aggregation of the NCs. Worth mentioning that the absorption spectrum looks mainly dominated by the LB medium compared to the Cu NCs spectrum.

The results obtained out of the UV-vis are further supported by the DLS results, as shown in the following figure the hydrodynamic size of the Cu NCs has no significant change after 24 h incubation with LB medium. Which supports the results obtained using optical spectroscopy.

Figure S9 UV-vis spectra of the Cu NCs vs LB broth medium at two different time points, 0 h and 24 h.

Figure S10 Hydrodynamic diameter (based on number distribution) of the Cu NCs vs LB broth medium at two different time points, 0 h, and 24 h.

# S4. Enzymatic-like activity of the NCs:

The artificial peroxidase-like activity of the NCs has been evaluated against the ortho-phenylenediamine (OPD) substrate. The catalytic activity of the NCs was tracked through their ability to oxidize the ortho-phenylenediamine (OPD) (colorless) into 2,3 -diaminophenazine (DAP) (yellow color). The oxidation process was traced using the UV-vis spectrophotometer by recording the evolution of the absorption spectra at λ = 425 nm, the characteristics band of DAP. [1]

Briefly, 100 µL of Cu NCs (A_λ400_ = 0.2) was mixed with 1.5 mL of OPD solution (0.5 mM) in different pH buffer media at 37 ^o^C. The evolution of the absorption spectrum of the oxidation product DAP has been recorded using a UV-vis spectrophotometer at different time points and the reaction kinetics was then determined.

Figure S11 Intrinsic oxidase-like activity of the Cu NCs. Kinetic OPD oxidation in the presence of Cu, time-dependent UV-vis spectra of DAP product, and absorbance changes at 425 nm upon OPD oxidation of 0.1 mM (A and B), 0.5 mM (C and D), and 1 mM (E and F).

Figure S12 Intrinsic Peroxidase-like activity of the Cu NCs. Kinetic oxidation of OPD in the presence of Cu NCs and 10 mM H_2_O_2_, time-dependent UV-vis spectra of DAP product, and absorbance changes at 425 nm upon OPD oxidation of 0.1 mM (A and B), 0.5 mM (C and D), and 1 mM (E and F).

## S5. Bacterial sensing of Cu NCs

**S5.1. Bacterial sensing of Cu NCs using OPD as an indicator**

The interaction between Cu NCs with Gram-negative and Gram-positive bacteria (Escherichia coli and Staphylococcus aureus, respectively) was tested using OPD as an indicator. Hydrogen peroxide was added as an oxidizing agent to accelerate the interaction. In a 96-well microtiter plate, 100 µL of each bacterial strain at concentrations ranging from 10^7^ to 10 colony-forming units per milliliter (CFU/mL) was added. Then, 50 µL of Cu NCs at concentrations of 12 and 24 µg/mL, along with 20 µL of 1mM hydrogen peroxide, were added to the plate. The plates were then incubated for 30 minutes at 37°C. After incubation, 50 µL of 10 mM OPD was added to the mixture, and the plates were incubated again for 30 minutes at 37°C. The absorbance was measured at 425 nm using a Tecan Spectro plate reader (INFINITE M PLEX). The percentage absorbance of OPD is demonstrated in Figure S13.

Figure S13 The percentage absorbance of OPD at$\lambda=425 nm$ against E. coli and S. aureus using H2O2 [1 mM] with A) 12 µg/mL and B) 24 µg/mL Cu NCs. *** p value < 0.001 and * p value < 0.05.


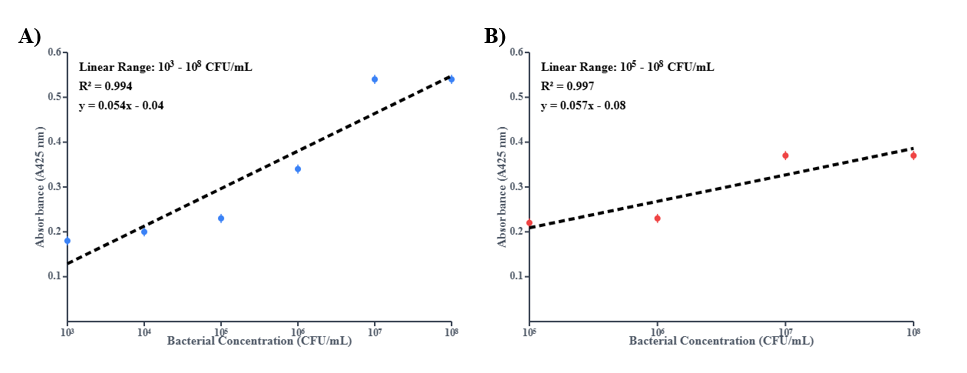


Figure S14 Calibration Curves Demonstrating the Linear Detection Range of Cu NCs for Bacteria. Calibration plots showing the change in absorbance (A425 nm) as a function of bacterial concentration (CFU/mL) for the Cu NCs biosensor. A) E. coli shows a linear detection range from 10^3^ to 10^8^ (CFU/mL), and B) S. aureus shows a linear detection range from 10^5^ to 10^8^ (CFU/mL).

**S5.2. The effect of Hydrogen peroxide on bacterial growth**

The impact of Hydrogen peroxide on the growth of both *E. coli* and S. aureus was examined (refer to Fig. 3). Each well of a 96-well plate was seeded with 100 µL of each bacterial strain at concentrations ranging from 10^7^ to 10^1^ CFU/mL. Subsequently, 20 µL of H_2_O_2_ (1 mM) was added to each well, and Luria-Bertani (LB) broth media was used as a control. The optical density (OD600) was measured using a Tecan Spectra plate reader after 24 hours of incubation at 37°C.

## S5.3. Quantitative antimicrobial activity of Cu NCs

The in vitro antibacterial activity of Cu NCs was tested on Gram-negative and Gram-positive bacteria, *Escherichia coli* and *Staphylococcus aureus,* respectively. The 50% inhibitory concentration (IC_50_) was analyzed using the broth microdilution method using vancomycin as a positive control. Briefly, a serial dilution of both Cu NCs and Vancomycin was added to 10^6^ CFU/mL of each bacterial strain in 96-well microtiter plates using Luria-Bertani (LB) broth medium. [2] The concentrations of Cu NCs ranged from 0 to 50 µg/mL, and vancomycin from 0 to 500 µg/mL. The plates were incubated at 37 ^o^C for 20 hours. After incubation, the optical density (OD600) was obtained using a TECAN Spectro plate reader. The IC_50_ values are shown in Table S2.

## S5.4. Statistical Analysis

The statistical analysis of the results was done using one-way analysis of variance (ANOVA) followed by Bonferroni’s Multiple Comparison Test as a post-hoc analysis to compare specific group differences using GraphPad Prism (GraphPad Software, Version 4.02, San Diego, CA, USA). Each sample was tested in triplicates, and the results were presented as the mean ± standard deviation. p < 0.05 indicating a significant difference.

**Table S2** IC_50_ of Cu NCs and Vancomycin against E. coli and S aureus.

| Sample | IC_50_ [µg/mL] | |
| --- | --- | --- |
|  | *E. Coli* | *S. aureus* |
| Cu NCs | 25 | 12.85 |
| Vancomycin | 250 | 62 |

# S6. References

1. Cursi, L., Mirra, G., Boselli, L. & Pompa, P. P. Metrology of Platinum Nanozymes: Mechanistic Insights and Analytical Issues. *Advanced Functional Materials,* 2315587 (2024).

2. Wiegand, I., Hilpert, K. & Hancock, R. E. W. Agar and broth dilution methods to determine the minimal inhibitory concentration (MIC) of antimicrobial substances. *Nat Protoc* **3,** 163–175 (2008).
